# The reverse motion illusion in random dot motion displays and implications for understanding development

**DOI:** 10.47691/joi.v3.7916

**Published:** 2022-01-10

**Authors:** Catherine Manning, Kimberly Meier, Deborah Giaschi

**Affiliations:** 1 School of Psychology and Clinical Language Sciences, University of Reading, UK; 2 Department of Experimental Psychology, University of Oxford, UK; 3 Department of Psychology, University of Washington, Seattle WA, USA; 4 Department of Ophthalmology & Visual Sciences, University of British Columbia, Vancouver BC, Canada

## Abstract

Across two independent developmental labs, we have been puzzled by the observation that a small proportion of our child and adult participants consistently report perceiving motion in the direction opposite to that presented in random-dot motion displays, sometimes even when the motion is at 100% coherence. In this review, we first draw together existing reports of misperceptions of motion direction in random dot displays across observers in a small percentage of trials, before reporting evidence of consistent reverse motion perception in a minority of observers, including previously unreported observations from our own studies of visual development. We consider possible explanations for this reverse motion illusion, including motion induction, motion energy, correspondence noise and spatial undersampling. However, more work is required to understand the individual differences relating to this percept. We suggest that errors in perceived motion direction are likely to be more widespread than can be currently gleaned from the literature and explain why systematic study is needed, especially in children. Finally, we list some remaining open questions and call for collaborative efforts to document this phenomenon and stimulate future investigation.

## 1 Introduction

Motion perception is an important function of the visual system that is fundamental to the control of eye movements and other actions, feeding into a range of other functions such as scene segmentation, depth perception and object recognition (Braddick, Atkinson & Wattam-Bell, 2003). Motion illusions, including illusions in which the perceived direction is reversed relative to the physical stimulus motion, have been reported widely. For example, reverse motion perception has been reported in complex gratings composed of different spatial frequencies (Derrington & Henning, 1987), noise patches containing both fine and coarse spatial frequency information (Serrano-Pedraza, Goddard & Derrington, 2007), patterns where the luminance contrast polarity reverses (‘reverse-phi’ motion, Anstis, 1970, and Nishida, 2011, for review) and extremely brief, large, high-contrast gratings (Glasser & Tadin, 2013; Glasser, Tadin & Pack, 2014). Occasional reverse motion percepts have also been reported in random dot patterns in a subset of trials across observers (Bae & Luck, 2018, 2019). Here, we review this evidence along with new evidence for a relatively unexplained and under-reported phenomenon: a small proportion of child and adult participants consistently perceive reverse motion in random dot motion stimuli. This occurs in some cases even at 100% dot coherence, and in participants with no known developmental or vision problems. We will refer to this consistent reverse motion perception as the reverse motion illusion, but related reports have referred to “opposite-direction perception”, “180° errors” (Bae & Luck, 2018, 2019), and “report of opposite direction” (Barbieri, Topfer, Soch, Bogler & Haynes, 2018). We consider possible explanations of the reverse motion illusion, including motion induction (from noise dots or static elements of the display), motion energy in the reverse direction, correspondence noise and spatial undersampling. However, these explanations remain incomplete until we can explain why only some observers experience consistently reversed motion perception.

The use of random dot motion stimuli is widespread because these stimuli are thought to tap global motion detectors, requiring extensive spatial and temporal integration over a range of frequencies (Bex & Dakin, 2002; Burr, Morrone & Vaina, 1998; Burr & Santoro, 2001, Yang & Blake, 1994; see review by Burr & Thompson, 2011). Moreover, these stimuli are commonly used to study the development of visual motion processing, revealing a protracted developmental trajectory across childhood (Hadad, Maurer & Lewis, 2011; Manning, Dakin, Tibber & Pellicano, 2014; Meier & Giaschi, 2017) and altered sensitivity in a range of developmental conditions (see Braddick et al., 2003, for review). As we will argue, it is therefore particularly important to consider the relevance of the reverse motion illusion for the study of visual development.

## 2 Previous reports of reverse motion perception for random-dot stimuli in a subset of trials

**Figure 1:**
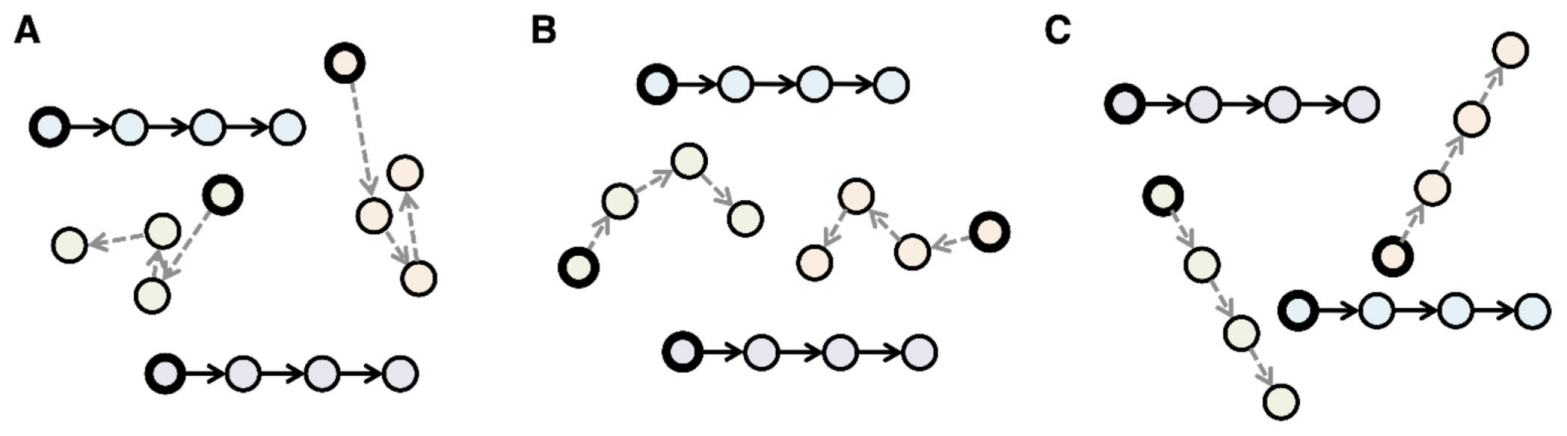
Schematic representations of common noise algorithms used in random dot stimuli. The dark border indicates where a dot is located on the first frame of the stimulus, and arrows indicate where a dot is moving on subsequent frames. In these examples, each dot remains a signal or noise dot for the duration of the stimulus (the ‘same’ signal selection rule as described by Scase et al., 1996), but signal and noise labels can also be assigned probabilistically on each frame (‘different’ rule). A. White noise algorithm, where noise dots are replotted with a random spatial offset in a random direction on each frame update. B. Brownian noise algorithm, where noise dots are replotted with a fixed spatial offset in a random direction on each frame. C. Transparent noise algorithm, where each noise dot is replotted with a fixed spatial offset in a fixed direction on each frame.

While not yet subject to a systematic investigation, reverse motion perception has been noted previously in adult participants in a minority of trials during coherent motion tasks. Coherent motion tasks require participants to discriminate the direction of signal dots moving among randomly moving noise dots (Newsome & Paré, 1988). The movement of noise dots is commonly controlled by one of three noise algorithms (Scase, Braddick & Raymond, 1996; see Figure 1): white (on each frame update, noise dots are replotted with a random spatial offset in a random direction), Brownian (noise dots are replotted with a fixed spatial offset in a random direction on each frame), and transparent noise (each noise dot is replotted with a fixed spatial offset in a fixed direction on each frame). The signal selection rule can also differ, with signal dots either remaining signal dots throughout the display sequence (‘same’ rule), or with signal dots being probabilistically re-selected on every frame (‘different’ rule, Scase et al., 1996). Typically, in a white noise stimulus, the ‘different’ rule is used, with each dot having a probability equal to the coherence level of being selected as a signal dot while the remainder are randomly replotted as noise dots. Conversely, in Brownian and transparent noise, the ‘same’ rule is typically used, where dots remain signal or noise throughout the trial, and are replotted in a random location when they reach the end of their ‘lifetime’. However, it is possible to use either rule with any noise algorithm, so a thorough description of stimulus properties is important for any study using this methodology.

Bae and Luck (2018, 2019) used a continuous motion report task where participants were asked to estimate the direction of 25% and 50% coherent motion stimuli (using white noise) from 0 to 360 degrees. They found two clusters of reports: one large cluster centered at the true direction, and one small cluster centered at 180 degrees of the true direction consisting of ~10% of trials (Bae & Luck 2019). This reverse motion report occurred on only a minority of trials, but across almost all participants. Similarly, in conference presentations, Barbieri et al. (2018) and Green and Pratte (2020) presented preliminary evidence of opposite motion direction reports, and additionally showed that these reports depended on stimulus parameters. Barbieri et al. found that the illusion was particularly apparent at lower coherence (12.5%, 25%, and 50%) transparent noise as opposed to Brownian noise stimuli, hypothesizing these reverse motion reports may be related to the ability to detect the correct motion axis but not discriminate the correct direction along that axis. Green & Pratte showed evidence of reverse motion reports, as well as reports ±90° from the signal direction. Interestingly, the misperceptions of direction they found were present in random dot motion stimuli comprised of separate distributions of signal and noise dots (as in Bae & Luck 2019 and Barbieri et al. 2018), but not in a Gaussian motion task where noise is added by increasing the standard deviation of the distribution around the signal direction from which the dot directions are sampled (e.g., Williams & Sekuler, 1984; Watamaniuk & Sekuler, 1992). This latter finding may suggest that reverse motion perception arises only where there is a requirement to segregate signal-from-noise rather than calculate an average motion direction.

Notably, all of the tasks described thus far have required participants to report perceived direction using a 0 to 360 deg method of adjustment. In the binary choice tasks (e.g., left vs. right; up vs. down) commonly adopted by researchers studying visual development and disorder, it is difficult to determine which incorrect responses are due to reverse motion perception unless the participant happens to describe their percepts, and so occasional reverse motion percepts may be obscured. Using a binary choice task, however, Blumenthal, Bosworth and Dobkins (2013) also provide the anecdotal observation that some adult participants occasionally experience reverse motion perception for stimuli at less than 100% coherence. To our knowledge, these reports have not yet been subject to a systematic investigation, perhaps in part because they reflect only a small percentage of trials. It will be important to understand what causes this illusory percept in these trials – whether it be a feature of the precise configuration of randomly placed dot stimuli on those trials, or a feature of the participant (e.g., attention levels or eye movements).

## 3 Previous reports of consistent reverse motion perception for random-dot stimuli (the reverse motion illusion) in a minority of observers

The particular phenomenon we have observed in our own studies, in contrast to the above, is *consistent* reports of reverse motion perception across trials in a minority of child and adult participants, and in some cases, *even for stimuli presented at 100% coherence*. Specifically, in binary choice discrimination tasks where participants are required to discriminate the direction of coherently moving dots, we have observed a small proportion of participants who perceive motion in the opposite direction, reflected in accuracy levels systematically *below* chance (at least until they correct their strategy in response to feedback). Before describing our own data, we will discuss two previous reports showing this consistent reverse motion illusion in a minority of observers. First, Morrone et al. (2008) reported that two of their 16 young patients (12.5%) (aged 10 and 16 years) with periventricular leukomalacia experienced systematic reverse perception of translational motion (but not rotation or expansion motion), specifically for random dot stimuli but not grating stimuli. The authors raised the intriguing possibility that the reverse motion illusion could be related to atypical brain development. However, the precise mechanisms are not clear, as the other patients with similar lesions did not experience the reversed motion illusion. Second, Dakin and Turnbull (2016) reported the reverse motion illusion in a small number of visually normal adult participants. In this study, adults continually monitored a fully coherent drifting (leftward/rightward) stimulus using large-field Gaussian noise patterns bandpass filtered for a range of spatial frequencies. They found that three of 30 observers (10%) performed significantly below chance when the stimuli were of high spatial frequency and low contrast. However, these opposite motion reports were not reflected in simultaneously-recorded optokinetic nystagmus responses. These reports show that there are individual differences in the likelihood of experiencing the reverse motion illusion.

## 4 New evidence of the reverse motion illusion for random-dot stimuli in a minority of adult observers

Throughout previous studies in our labs, we have observed additional evidence for the reverse motion illusion in a minority of observers. We have noted two points of particular interest. First, the reverse motion illusion is seen even in participants without any known developmental or vision problems, and second, the reverse motion illusion is sometimes perceived even at 100% coherence. The first time one of the authors noticed certain observers experiencing the reverse motion illusion was in 1998 when collecting data for a study involving participants with dyslexia (Edwards et al., 2004). We have since noted the phenomenon in neurotypical adults. For example, in a study by Meier & Giaschi (2014) using white noise stimuli (‘different’ rule) and left/right discrimination, 2 of the 42 recruited adults (4.8%) perceived motion in the opposite direction to the coherent motion direction at high coherence (see Videos 1 and 2 for example stimuli). The participants with anomalous perception had normal visual acuity and stereoacuity, and no known clinical conditions. In a more recent study using an identical paradigm (Meier & Giaschi, 2019), one out of the 29 recruited adult participants (3.4%) gave responses which appeared to be reversed for 100% coherence stimuli, specifically for stimuli with large spatial displacements (Δx = 30 arcmin). In an unpublished dataset collected for a Master's thesis (Meier, 2013) 3 of 25 adults (12%) experienced the reverse motion illusion for Brownian motion (‘same’ rule).

In addition to adults with healthy vision, we have also noted the reverse motion illusion in at least one adult with strabismus (tested but not included in the study reported by Meier, Spering & Giaschi, 2019) who had normal visual acuity, but no measurable stereoacuity. This participant saw reverse motion for high coherence stimuli, specifically for fast stimuli. Analysis of eye position data as a function of trial accuracy indicated that for high coherence stimuli, this participant made OKN-like drift responses consistent with the signal direction of the stimulus, even when reporting a response in the opposite direction, similar to Dakin & Turnbull (2016). Together, these studies highlight that we need to explain why some individuals appear to consistently experience the reverse motion illusion in motion displays. It is possible that individual differences originate during development. We therefore now turn to evidence for the effect in children.

## 5 New evidence of the reverse motion illusion for random-dot stimuli in a minority of child observers

**Figure 2:**
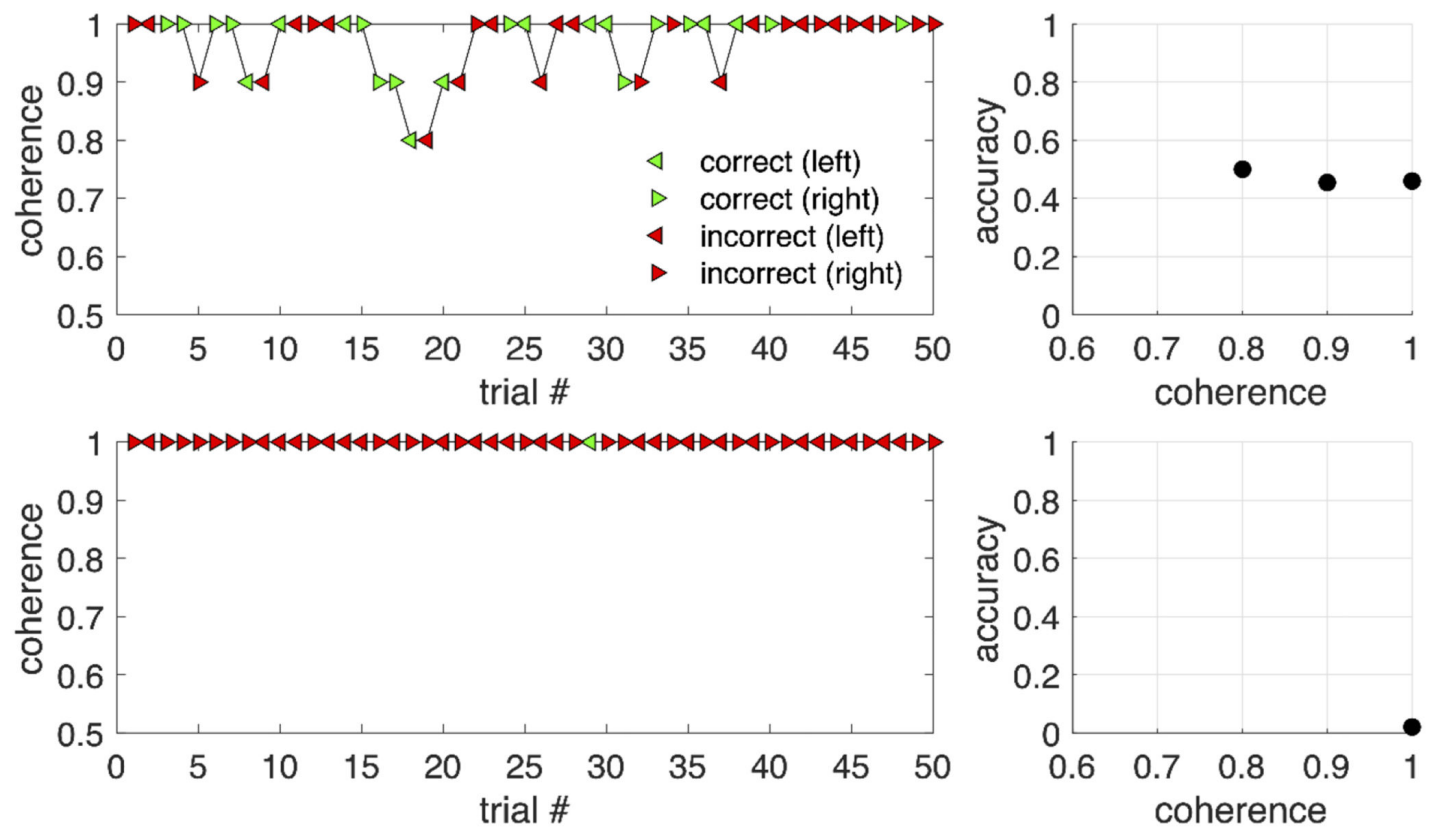
Data from two control children collected as part of (but not included in) a study of typical development (Meier & Giaschi, 2017) in which stimulus coherence was controlled by a 2-down 1-up staircase with 50 trials. Left panels show trial-by-trial responses of these children, and right panels show average accuracy as a function of coherence. Feedback was given to participants after each trial, and both children reported to research assistants that they were confused by the feedback because they were seeing motion in the opposite direction. The 10-year-old participant shown in the top row attempted to adapt to the feedback by changing which button they pressed, but still only achieved near-chance level performance. The 7-year-old participant shown in the bottom row did not change their strategy in response to feedback, and showed consistently below-chance performance at 100% coherence. In both cases the staircase did not advance to lower coherence levels. These data highlight that the reverse motion illusion may not be obvious by simply looking at average accuracy, especially when using staircases with feedback.

Here we present observations from our own studies which show that the reverse motion illusion is seen in a small proportion of children, including those without any known developmental or vision problems. In addition to the two adult participants who experienced reverse motion in Meier & Giaschi (2014) reported above, 2 of the 33 children aged 4 to 7 years recruited for this study (6.1%) also saw reverse motion at the beginning of the test session. These participants responded to feedback in practice trials so that they were able to give correct answers for the remainder of the test session. Similarly, 1 of the 33 children recruited for Meier & Giaschi's (2019) study (3.0%) also perceived reverse motion. A further study using the same stimuli in children with amblyopia and typically developing controls (Meier & Giaschi, 2017; Meier, Sum & Giaschi, 2016) found 6 control children out of 217 (2.8%) who perceived reverse motion (see Figure 2).

While these studies presented stimuli with a white noise algorithm, we have also found evidence of the reverse motion illusion in children using a Brownian noise algorithm (see Video 3 for example stimuli). Using this type of random dot motion stimulus with limited lifetime dots (‘same’ rule; 3% of dots replotted every 40 ms), Narasimhan and Giaschi (2012) reported that 4 of their 11 child participants (36.4%) aged 5 to 6 years perceived motion in the reverse direction, with the experimenter inverting the response pad so that they could continue the experiment. One of these participants was also a participant who saw the reverse motion illusion in the studies reported by Meier & Giaschi (2017) and Meier et al. (2016). In an unpublished dataset investigating motion coherence thresholds in dyslexia using a Brownian noise algorithm, 2 of 21 children with dyslexia (9.5%) and 3 of 22 children without reading difficulties (13.6%) perceived the reverse motion illusion. In a further unpublished dataset of dyslexic participants and their family members (n = 136 in total), we found one child with dyslexia and one adult and four child controls who perceived the reverse motion illusion (4.4% of the sample).

**Figure 3:**
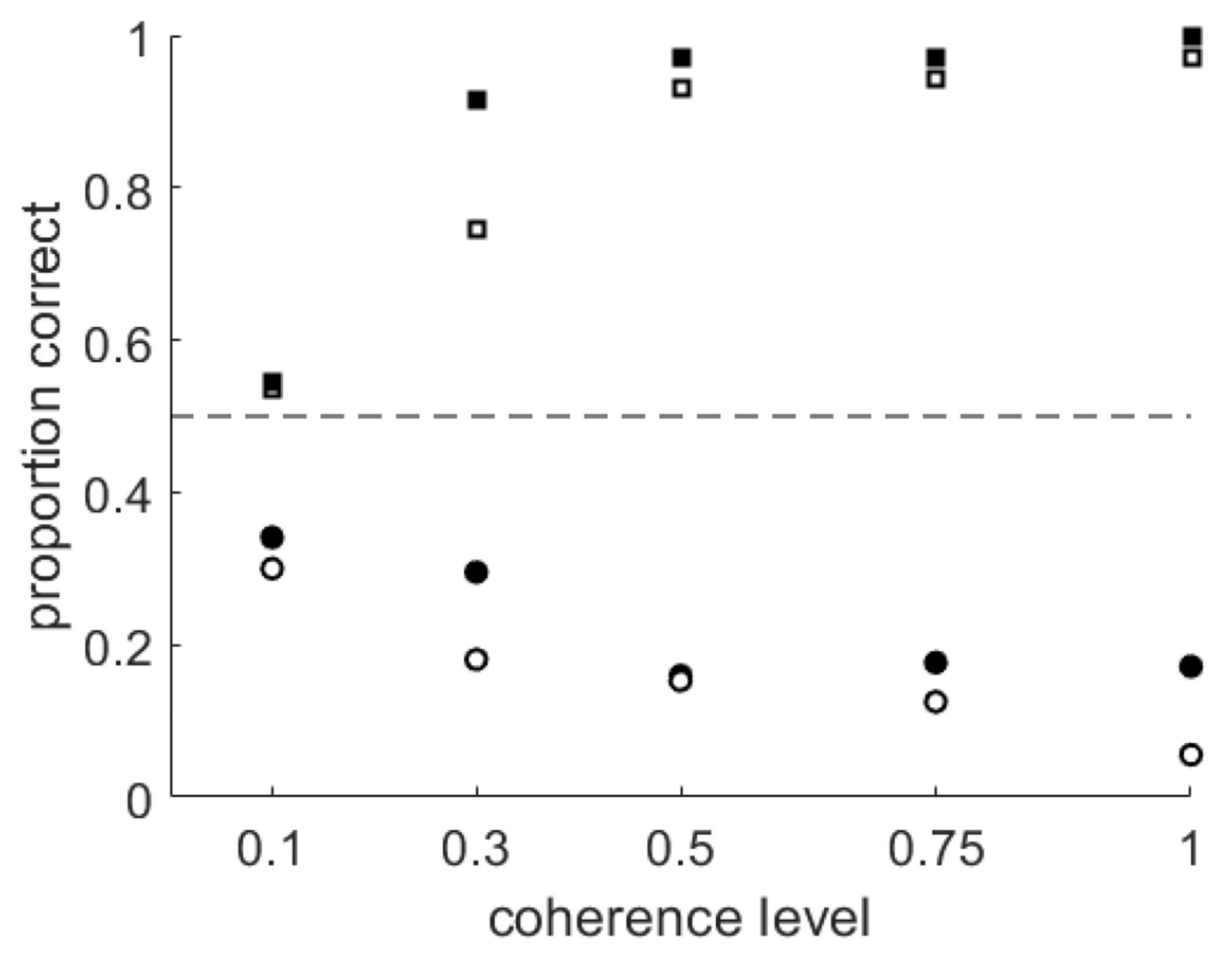
Data belonging to two child participants from Manning et al.'s (2019, 2021) study who showed below-chance accuracy at all levels of coherence (circles) and, for comparison, two child participants who responded as expected (squares). In this task, participants completed a binary choice coherent motion direction discrimination task. Performance that is systematically below-chance (indicated by the horizontal dotted line) therefore indicates the reverse motion illusion, which was corroborated with the participants' verbal comments. See Video 4 for an example of a stimulus with .75 coherence.

In a dataset reported by Manning and colleagues (Manning, Kaneshiro, Kohler, Duta, Scerif & Norcia, 2019; Manning, Wagenmakers, Norcia, Scerif & Boehm, 2021) which used a transparent noise algorithm with a ‘same’ signal selection rule, 16 out of 109 children aged between 6 and 12 years (14.7%) explicitly reported seeing motion in the opposite direction or gave responses consistent with this percept in a coherent motion task (see Video 4 for an example of the stimuli). Differences in this task compared to that used by Giaschi and colleagues were that the coherent motion stimulus was preceded by a completely random motion stimulus to facilitate the recording of visual evoked potentials to directional motion onset, and that the signal dots moved either upwards or downwards (as opposed to leftwards/rightwards). Moreover, the lifetime of the dots was limited to 200 ms and the stimulus was presented until the participant made a response. The participants who experienced the reverse motion illusion with these stimuli all had binocular visual acuities in the normal range, and no diagnosed developmental conditions. Most of these participants responded to feedback so that they responded with above-chance accuracy in the main experimental trials, although 2 participants who were siblings continued to give responses in the opposite direction across all levels of coherence (see Figure 3) and were excluded from behavioural analysis (Manning et al., 2021). Interestingly, none of the 20 adults tested on this paradigm reported perceiving the reverse motion illusion or gave responses consistent with reverse motion perception. Collectively, these rates indicate that children may be more likely to experience the reverse motion illusion than adults, but more intentional documentation of this phenomenon is needed to confirm this.

**Figure 4:**
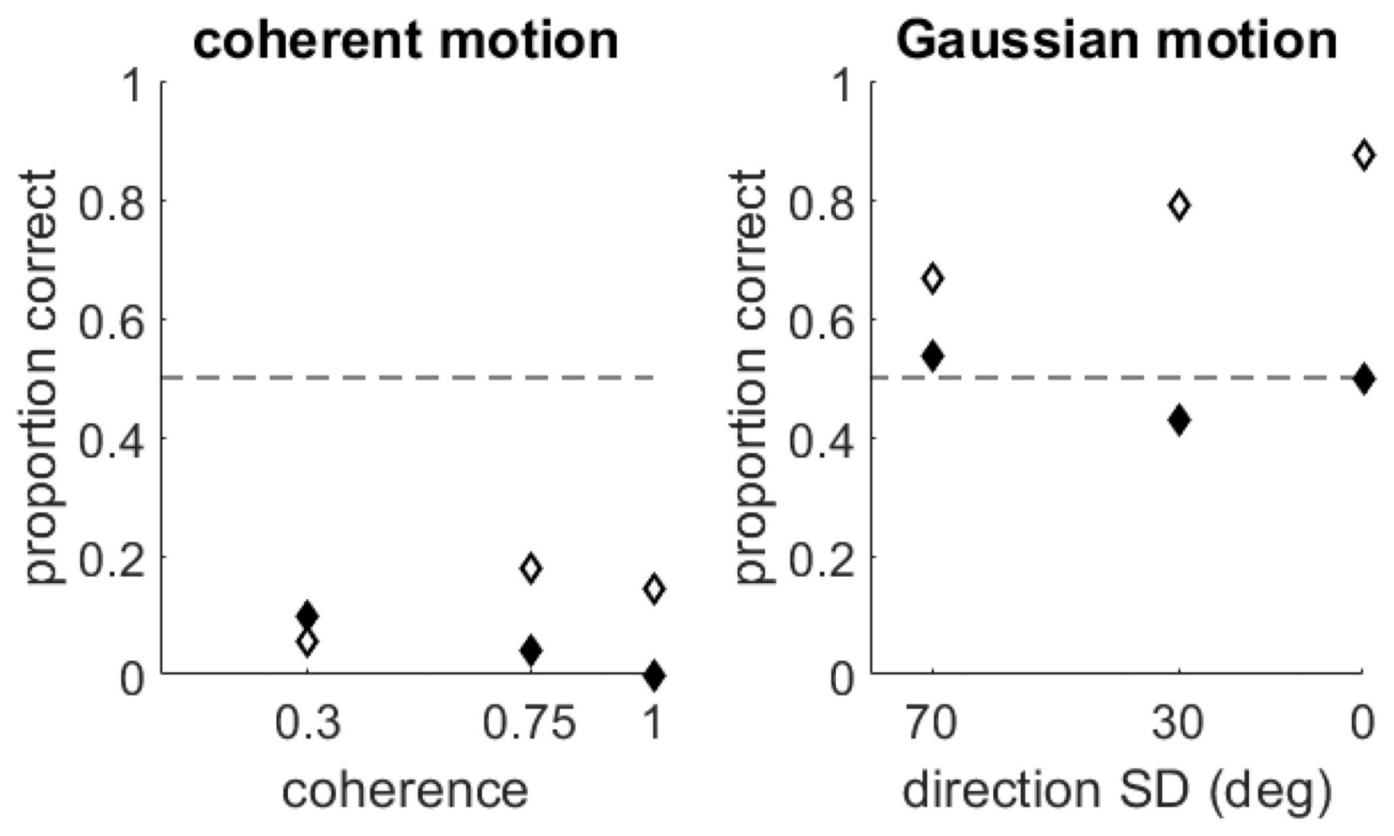
Data belonging to two child participants with dyslexia from Toffoli et al. (2021) who had performance that was systematically below chance in a coherent motion task (left). In a Gaussian motion task (right), one of these participants performed near chance (black diamonds), whereas the other performed above chance (white diamonds). Chance-level performance is indicated with a horizontal, dashed line. Importantly, the stimuli presented in 100% coherence trials for coherent motion are equivalent to the trials with 0 deg standard deviation of directions in the Gaussian motion task. This therefore suggests that the children were using a different strategy in the two tasks.

A modified version of Manning et al.'s (2019, 2021) task was presented to typically developing children and children with an autism or dyslexia diagnosis aged between 6 and 14 years by Toffoli and colleagues (Toffoli, Scerif, Snowling, Norcia & Manning, 2021). In this study, some changes were made to the task with the aim of minimizing the reverse motion illusion in children. Specifically, dot lifetime was increased to 400 ms, the direction of coherent motion was leftward or rightward and an auditory tone was presented at the same time as the transition between random and coherent motion to eliminate temporal uncertainty about the stimulus onset. Additionally, participants completed an additional direction discrimination task whereby the dot directions were taken from a Gaussian distribution on each trial (cf. Green & Pratte, 2020; Watamaniuk & Sekuler, 1992). Out of the 185 children tested for this study, 9 children (4.9%; 5 typically developing, and four with a dyslexia diagnosis) reported seeing motion in the opposite direction. This is a relatively smaller proportion than the participants who perceived the reverse motion illusion in the study by Manning et al. (2019, 2021), which could be attributable to differences in the stimulus parameters. Interestingly, for a couple of these children, the reverse motion illusion appeared to occur only for coherent motion stimuli rather than Gaussian motion stimuli (Figure 4), reflecting Green & Pratte's (2020) findings from a continuous motion report task in adults. However, some children showed the reverse motion illusion even for catch trials in which all dots moved in the same direction (100% coherence, or 0° standard deviation), similar to participants from Meier & Giaschi (2014, 2017, 2019) and Meier et al. (2019) who reported backward direction on 100% coherence trials, suggesting that the reverse motion illusion is not fully attributable to the presence of randomly moving noise dots across all observers.

## 6 Explanation of the reverse motion illusion in random dot stimuli

There are a range of explanations that may account for the reverse motion illusion in random dot stimuli. First, the illusion could result from motion induction, whereby the signal dots make the noise dots appear to move in the opposite direction (Blumenthal et al., 2013). Indeed, a comment from one participant in Manning et al.'s (2019, 2021) study suggested that they were experiencing motion induction and using a compensatory strategy in order to provide the correct response: “When most of them are going in the same direction, I look at where the others are going and press the opposite”. Motion induction could be particularly common at mid-to-low levels of coherence, as the independent populations of signal and noise dots could lead to the percept of two competing surfaces sliding across each other. The noise dots may be more visually salient than the signal dots, as they stand out against an otherwise uniform display. Anecdotally, participants (and research assistants) in studies from the Giaschi lab have reported seeing dots move in both directions at mid-level coherences for stimuli using Brownian, but not white, noise algorithms. We assume this is because Brownian noise creates a “wiggly” noise surface that is induced to move in the opposite direction of the signal dots, and so participants report on the direction of the induced rather than the inducing motion; the random replotting of white noise dots does not create such a surface.

Additionally, a few participants in Manning et al.'s (2019, 2021) study said things like “I'm looking at the ones at the back”, despite no depth information being presented. Two competing surfaces moving in opposite directions can also cause a person to perceive the surfaces at different depths (see Qian, Anderson & Adelson, 1994), and if participants interpret signal and noise dots as competing surfaces, this may strengthen the motion induction and contribute to the reverse motion illusion. Relative number, speed, and density of the dots in each surface can all impact perceived depth order (Moreno-Bote, Shapiro, Rinzei & Rubin, 2008; Schütz, 2011), often idiosyncratically (Hwang & Schütz, 2020), and these factors can all vary as a function of stimulus coherence level.

However, we do not think that this is the entire explanation of the reverse motion illusion, as more surprisingly, some participants experienced the illusion even at 100% coherence when there were no noise dots (see Figures 2-4). Yet stationary aspects of the display, such as the fixation point, could also be affected by motion induction. Consistent with this, some people in our studies have reported that the fixation point appears to move in the direction opposite to the signal dot direction. For example, in the study by Manning et al. (2019, 2021), one child said: “When the red thing [fixation point] is going up it's [the dots] going down”, and additionally, “the red thing only moves on some of them [trials] and its very confusing”. To overcome this motion induction cue when it appeared in children and adults tested by Meier and Giaschi (2014; 2017) and Meier et al. (2016), research assistants would conduct practice trials while using a stick or a finger to point at the fixation cross to reduce the strength of motion induction, or following the path of moving dots with a finger or pencil - techniques that worked to eliminate opposite direction reports in some participants prior to conducting the experiment. Participants who were successfully trained to discount motion induction cues in this way are not counted as having a reverse motion illusion in our descriptions above, and were always capable of successfully completing practice trials. Removing the fixation point also appeared to reduce the experience of reverse motion for some observers, though not all. Future studies could therefore systematically investigate the effect of removing the fixation point on the occurrence of the reverse motion illusion to rule out motion induction as an explanation.

**Figure 5:**
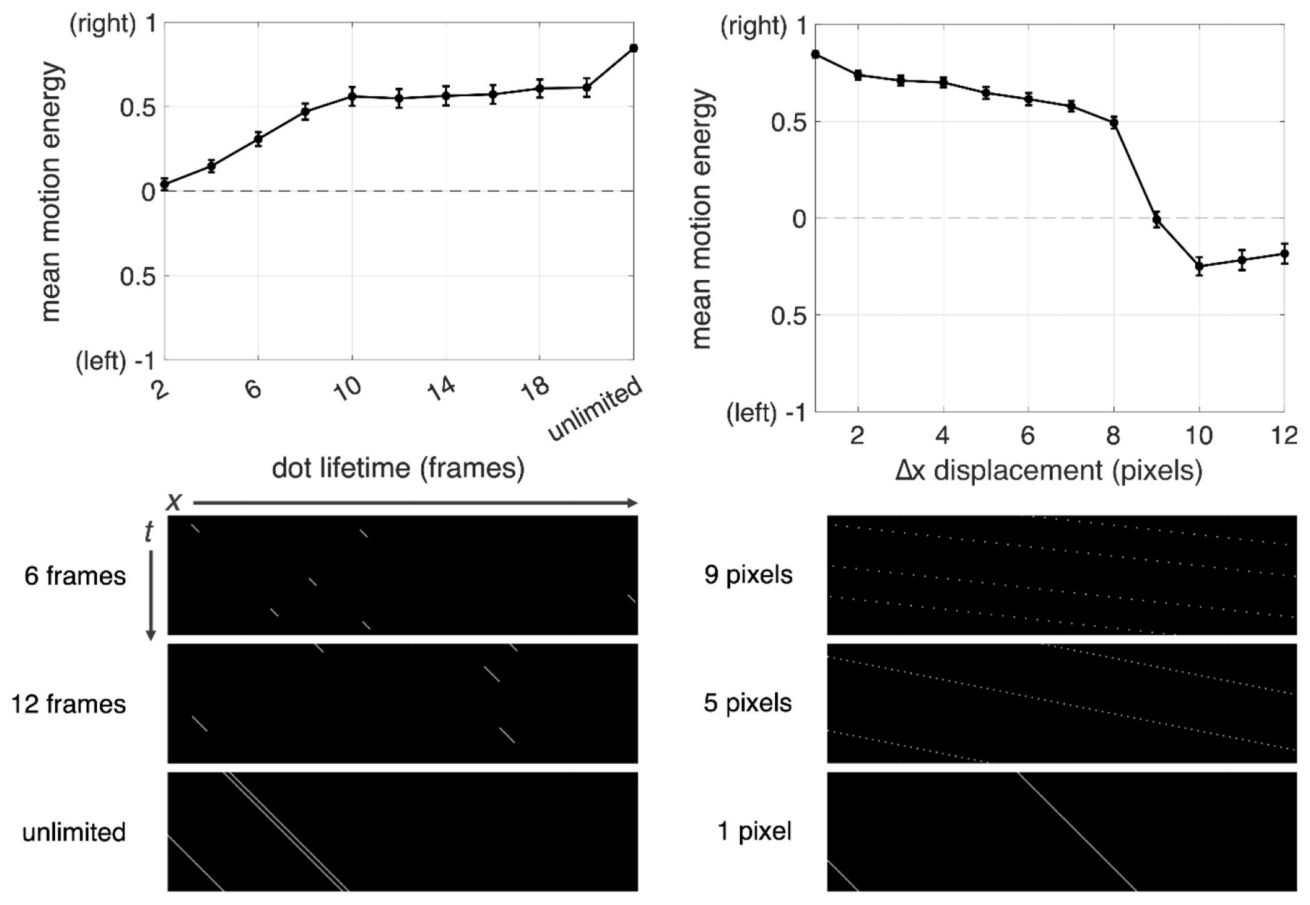
Top: Normalized motion energy as a function of signal dot lifetime (left panel) and spatial displacement (right panel) in a white noise global dot motion stimulus. Each data point reflects the mean and standard error for energy calculated across 100 (*X, t*) stimulus matrices randomly generated for each condition. Bottom: Example (*X, t*) plots showing stimuli that vary as a function of lifetime (left panel) and of spatial displacement (right panel). These plots depict the position (*X*) of a one-pixel dot as a function of time (*t*) for one horizontal “slice” of a dot motion stimulus.

Another possibility is that the illusion of reverse motion occurs when a stimulus contains motion energy (Adelson & Bergen, 1985) in opposing directions. This can occur when motion in the same direction at two different spatial scales in the same stimulus ‘cancel’ each other out to generate motion energy that is stronger in the opposing direction (Serrano-Pedraza et al., 2007). A 100% coherent stimulus with unlimited dot lifetime may predominantly contain motion energy in a single direction, but a number of stimulus parameters can impact the degree to which energy in the opposite direction exists. To demonstrate, Figure 5 shows relative motion energy^1^ normalized to total energy for rightward stimuli presented at 100% coherence as a function of dot lifetime and dot displacement (Δx). For example, when dot lifetime is limited to two frames, energy is only slightly greater for the rightward than for the leftward direction; but as dot lifetime increases, energy in the rightward signal direction dominates. Similarly, when lifetime is unlimited and Δx is increased (equivalent to increasing dot speed), energy in the rightward signal direction decreases relative to the leftward direction, even to the point where leftward energy begins to dominate. Note that our model calculates motion energy at only one spatial scale, and using a model with a smaller spatial frequency filter (or a multi-scale model incorporating lower frequency bands) is more robust against this reversal of direction in motion energy calculations, at least at 100% coherence.

While this simple motion energy model is a theoretical demonstration, there is evidence that our brains are sensitive to this information. For example, Nishida and Sato (1992) found a motion after-effect in the same direction as signal dots, likely induced by adaptation to motion energy in the opposite direction. In line with this account, Chetverikov & Jehee (2019) decoded the posterior probability of motion direction from activity in visual areas V1-V4 and hMT+ and uncovered bimodal distributions, one with a larger peak centered on the true motion direction, and the other with a smaller peak centered on the opposite direction. A neural network (MotionNet) trained on moving images showed responses in the opposite motion direction, particularly for MT units which prefer faster speeds (Rideaux & Welchman, 2020, see their Figure 5c).

The reverse motion illusion could also be influenced by correspondence noise (Barlow & Tripathy, 1997). This term describes false correspondences between dot pairs on successive frames, and the likelihood of these mismatches can increase as stimulus coherence decreases. Correspondence noise can also be introduced by the use of limited lifetime dots, and as a result these spatiotemporal pairings can generate motion energy in all directions, including the direction opposite to the signal direction even for stimuli at 100% coherence (as in Figure 5, above). While correspondence noise should not necessarily lead to a reverse motion illusion, the decreased signal strength may lead to increased uncertainty and a higher probability of an incorrect response even for high coherence stimuli. For example, with limited lifetime dots some children reported seeing “jumpy” motion, which we think was caused by a proportion of dots decaying and being replotted in new locations on each frame (Manning et al. 2019, 2021; Toffoli et al., 2021). One child said that they were pressing the ‘up’ key if the stimulus dots were “jumping down”. Alongside other task differences, the study by Toffoli et al. (2021) lengthened the limited lifetime duration compared to Manning et al. (2019, 2021), which could have contributed to a smaller proportion of participants experiencing the reverse motion illusion in this later study. Notwithstanding the need for more systematic evidence, one child participant who experienced the reverse motion illusion with limited lifetime stimuli in Manning et al.'s (2019, 2021) study did not show this illusory percept when the lifetime was extended.

Sensitivity to motion energy in non-signal directions may also be related to aliasing as a result of spatial undersampling. This explanation was proposed by Morrone et al. (2008) in the study of periventricular leukomalacia patients, and echoes the explanation for reverse motion percepts in complex grating patterns made by Derrington & Henning (1987; see also Coletta, Williams & Tiana, 1990). Undersampling may have the same effect on motion energy as increasing the spatial displacement between frames (see Figure 5), and therefore increase the likelihood of reverse motion percepts.

Yet unanswered by all of these accounts is why we observe the reverse motion illusion in only a small proportion of participants. Perhaps there are individual differences in neurophysiology that interact with stimulus parameters. For example, Morrone et al. (2008) reported that translational motion did not activate motion-sensitive area hMT+ in their participants who experienced the reverse motion illusion, and suggested that this was due to damage to inputs to motion-selective neurons which led to spatial undersampling and, as a result, aliasing of motion direction. It would be interesting therefore to see whether a similar explanation can be put forward for children without developmental conditions who experience the reverse motion illusion. There may also be individual differences in attention or eye movements that explain this difference. In support of this suggestion, Morrone et al. (2008) reported that the two participants who experienced the reverse motion illusion in their study also had difficulties tracking targets. However, Dakin and Turnbull's (2016) study suggested a dissociation between perceived motion direction and optokinetic eye movements. Similarly, our strabismic participant reported above (Meier et al., 2019) made eye movements consistent with the signal motion direction regardless of the direction they reported.

## 7 An under-reported phenomenon?

Until now, we have not discussed our observations of reverse motion perception by a minority of observers in our published studies, apart from sometimes mentioning the numbers of participants who have been excluded from further analysis. However, following informal exchanges with other scientists at conferences and on social media, we believe that this phenomenon may be more widely spread than is immediately apparent from the literature. While the data from participants perceiving the reverse motion illusion are often excluded from further analysis, these data could be telling us something important about individual differences in motion perception. Additionally, it is a shame to discard data, particularly data belonging to children, for whom psychophysical data are relatively time-consuming to obtain. In this section we consider why ignoring the reverse motion illusion (particularly in studying development) may be problematic.

Confusion caused by the reverse motion illusion could be greater in children than in adults, leading to a relative underestimation of children's sensitivity to coherent motion (as suggested by Blumenthal et al., 2013). Alternatively, even if children are not more confused than adults, adults may be more willing to verbalise their confusion, meaning that more participants experiencing the reverse motion illusion will be excluded from adult datasets than child datasets. It is possible that this confusion (or unwillingness to verbalise it) could partially contribute to the slow development of motion sensitivity that is revealed by random dot motion stimuli (e.g., Hadad et al., 2011; Manning et al., 2014) and potentially, to differences between typically and atypically developing children (e.g., Milne, Swettenham, Hansen, Campbell, Jeffries & Plaisted, 2002; Pellicano & Gibson, 2008). Moreover, if young children are not always able or willing to verbalise that they are experiencing motion in the reverse direction, the effect may be more widespread among children than can be currently known. Additionally, participants may adjust their strategy in response to feedback. Indeed in our studies, some of the participants who initially indicated seeing motion in the opposite direction were able to use feedback to change their strategies so that they were able to perform at a good level of accuracy in the task, and we were aware of this due to verbal comments they made. For example, one child from the Manning et al., (2019, 2021) study said: “so when it looks like it's going up, it's going down”. For these children, the task then becomes more of an inhibitory control task, where they must suppress their prepotent response, and thus performance might be limited by executive functioning skills independent of motion perception abilities per se. Therefore, the processes underlying performance might vary across children, making it difficult to interpret their performance as a homogenous group.

Some developmental studies use coherent motion tasks that do not require direction report (e.g., studies requiring participants to detect a patch of coherent motion, Atkinson, King, Braddick, Nokes, Anker & Braddick, 1997; Braddick et al., 2016, 2017; Gunn et al., 2002; Manning, Charman & Pellicano, 2013; or discriminate shapes formed by motion contrast, Giaschi & Regan, 1997; Hayward, Truong, Partanen & Giaschi, 2011; Parrish, Giaschi, Boden & Dougherty, 2005). In these paradigms, participants can do well even if they perceive motion in the opposite direction, so a reverse motion illusion can go unnoticed. Crucially, this could lead to a divergence in apparent thresholds obtained from coherent motion detection and direction discrimination tasks. It is also worth reflecting that random dot motion displays are often used with non-human primates who have been trained to respond correctly in response to feedback (e.g., Britten, Shadlen, Newsome & Movshon, 1992). Here, it is possible that motion is perceived in the opposite direction to the stimulus motion, but the researcher has no way of knowing, and potentially important individual differences in motion processing are being missed.

## 8 Open questions

We have reviewed evidence that a small proportion of both child and adult observers consistently report motion in the opposite direction to the stimulus direction in random dot motion displays, and in some cases at 100% dot coherence. We have suggested a range of potential explanations, but more work is needed to understand the source of individual differences. We have also considered the implications of this effect for properly characterizing visual development. One open question relates to the stimulus parameters that most strongly elicit the reverse motion illusion. This question is difficult to address based on the current data, as there are many differences in how random dot motion stimuli are implemented, and different parameters can have a large impact on psychophysical performance (Meier & Giaschi, 2014; Narasimhan & Giaschi, 2012; Pilly & Seitz, 2009; Scase et al., 1996) as well as eye movement characteristics (Schütz, Braun, Movshon, & Gegenfurtner, 2010). Moreover, not all studies provide a full and complete description of stimulus characteristics. It is therefore difficult to compare findings across studies and isolate the most important factors. As such, we stress the importance of reporting stimulus details in full, including Δx and Δt displacements (not just speed), the specific algorithm used to displace noise dots, and whether or not signal/noise dots are reassigned probabilistically on each animation frame (‘same’ vs ‘different’ rule). Moreover, keeping a record of participants who experience the reverse motion illusion is key, so they can be invited back for additional research. Future studies could assess the importance of each stimulus parameter, while holding others constant. There are at least three candidate parameters that we think warrant further investigation. First, the reverse motion illusion may be found particularly for shorter vs. longer (or unlimited) dot lifetimes, as suggested above, and second, for stimuli with larger dot displacements. Third, it is possible that the reverse motion illusion might be more commonly found for random dot stimuli using a ‘same’ signal selection rule (where the signal and noise dots stay the same throughout the display sequence) rather than a ‘different’ signal selection rule (where signal and noise dots are reassigned throughout the display sequence), as the ‘same’ signal selection rule may lead to greater segregation between signal and noise dots, making induced reverse motion in the noise dots more visible. Systematic research could determine which are the best stimulus parameters to use in order to avoid the reverse motion illusion.

Another open question is whether we should be looking for a single explanation, or whether there may be different explanations for different participants and stimulus configurations. A related question is whether the occasional report of opposite direction in continuous report tasks (e.g., Bae & Luck, 2019; Green & Pratte, 2020) is linked to our findings of consistent reversed motion perception in a minority of participants. Systematic study comparing participants' performance across tasks would help to address this. Specifically, it would be interesting to know whether participants who systematically report the reverse motion illusion in a binary choice task are also those who show more judgments clustering around ±180 deg of the stimulus direction in continuous motion report, and if the same stimulus parameters are critical in both cases.

To determine the developmental underpinnings of the effect in adults we will need to ascertain how stable the effect is across developmental time, using longitudinal studies. We know of one child who perceived the reverse motion illusion in Narasimhan & Giaschi (2012) at 5 years of age who also perceived the illusion when participating in the study by Meier et al. (2016) almost 4 years later, at age 9. However, we did not note recurring reverse motion percepts in three other participants returning to the lab. If the reverse motion illusion affects younger children more than older children and adults, this could be contributing to elevated motion coherence thresholds in children and a dependence on stimulus parameters (e.g., Meier & Giaschi, 2014; Meier et al., 2016; Narasimhan & Giaschi, 2012). It would also be informative to determine if there might be genetic factors explaining why some participants experience the illusion. Remarkably, we have noted siblings who similarly experience the illusion, including a sibling pair tested by Meier et al. (2016), a sibling pair tested by Manning et al. (2019, 2021, data in Figure 3), and notably a set of 4 siblings (2 of whom were twins) aged between 11 and 16 years, who all experienced the illusion in an unpublished dataset of dyslexic probands and their family members. However more data are needed to understand whether siblings show the same behaviour due to shared genetic or environmental influences, chance, or perhaps something about the testing situation on the day that the siblings are seen. Moreover, we need to investigate whether the reverse motion illusion is more common in atypically developing samples than typically developing samples, as motion processing difficulties have been reported in a range of developmental conditions (Braddick et al., 2003). Answering these questions will help to understand the mechanisms behind the reverse motion illusion.

Finally, while addressing the questions listed here will increase our scientific understanding of motion processing in both typical and atypical development, arguably the most important question is whether this illusory percept has any implications for real-world perception. While it is likely this phenomenon is restricted only to certain stimulus parameters relating to artificial random dot motion displays, especially since participants do not anecdotally report experiencing problems with motion perception, it could be indicative of visual functioning difficulties that also hinder daily perceptual tasks. Therefore, studying performance in random dot motion stimuli alongside more ecologically valid perceptual tasks is vital.

## 9 Call for collaboration

People who systematically experience the reverse motion illusion in random dot motion stimuli are rare, with the data that we present here being collected over a long time across a range of different studies and labs. Therefore a widespread, collaborative effort is needed to understand this phenomenon. If you have noticed this happening in your previous studies, please get in touch so that we can better characterise the participant and stimulus parameters that may result in this illusion. Ultimately, we see the value in a collaborative study across labs where participants who experienced the illusion are reinvited to take part in a more systematic study. We also suggest reporting information about participants who experienced the reverse motion illusion in forthcoming publications to help assess the extent of the phenomenon. In the case that participants do experience reverse motion perception, it may be possible to try manipulating the display to investigate whether they are experiencing motion induction or being affected by false correspondences, by seeing how performance changes if the fixation point is removed, or if the dot lifetime is extended. It is important to work sensitively with participants, particularly child participants, so that they do not get upset if they perceive the reverse motion illusion and get the answers ‘wrong’. Reassuring participants that we know that other people perceive motion in the reverse direction may help in some cases.

For those who use random dot motion displays but have not encountered this phenomenon before, we urge you to be alert to this issue. It may be that you have not experienced this personally because research assistants have been administering the tasks to participants. We note also that adaptive methods used to present coherence levels may mean that low coherence levels are not presented to a participant if they report the direction incorrectly at high coherence levels, and the participant may be excluded on the basis of being unable to do the task or being flagged as having a deficit. In these cases, it is particularly valuable to talk to participants to determine whether they are seeing reverse motion. We hope that this article will stimulate future, collaborative efforts to investigate this important phenomenon, with implications for studying the development of visual motion perception.

## Supplementary Material

Video 1

Video 2

Video 3

Video 4

Video caption

## Footnotes

1 Code to generate these models was adapted from George Mather, who provides a helpful demo at www.georgemather.com/Model.html. Spatiotemporal filter parameters were based on previous work (detailed in Challinor & Mather, 2010); spatial: *f* = 1.1 cpd, σ = 0.5 deg; temporal: k = 100, n = 6 and 9.

## References

[R1] Adelson EH, Bergen JR (1985). Spatiotemporal energy models for the perception of motion. Journal of the Optical Society of America A.

[R2] Anstis S (1970). Phi movement as a subtraction process. Vision Research.

[R3] Atkinson J, King J, Braddick O, Nokes L, Anker S, Braddick F (1997). A specific deficit of dorsal stream function in Williams’ syndrome. Neuroreport.

[R4] Bae GY, Luck SJ (2018). Motion perception in 360 degrees. Journal of Vision.

[R5] Bae GY, Luck SJ (2019). Decoding motion direction using the topography of sustained ERPs and alpha oscillations. NeuroImage.

[R6] Barbieri R, Töpfer F, Soch J, Bogler C, Haynes JD (2018). Feature-continuous motion judgements: Assessing different random dot motion displays. Journal of Vision.

[R7] Barlow H, Tripathy SR (1997). Correspondence noise and signal pooling in the detection of coherent visual motion. Journal of Neuroscience.

[R8] Bex PJ, Dakin SC (2002). Comparison of the spatial-frequency selectivity of local and global motion detectors. Journal of the Optical Society of America A.

[R9] Blumenthal EJ, Bosworth RG, Dobkins KR (2013). Fast development of global motion processing in human infants. Journal of Vision.

[R10] Braddick O, Atkinson J, Akshoomoff N, Newman E, Curley LB, Gonzalez MR (2017). Individual differences in children’s global motion sensitivity correlate with TBSS-based measures of the superior longitudinal fasciculus. Vision Research.

[R11] Braddick O, Atkinson J, Newman E, Akshoomoff N, Kuperman JM, Bartsch H (2016). Global visual motion sensitivity: Associations with parietal area and children’s mathematical cognition. Journal of Cognitive Neuroscience.

[R12] Braddick O, Atkinson J, Wattam-Bell J (2003). Normal and anomalous development of visual motion processing: motion coherence and ‘dorsal-stream vulnerability’. Neuropsychologia.

[R13] Britten KH, Shadlen MN, Newsome WT, Movshon JA (1992). The analysis of visual motion: a comparison of neuronal and psychophysical performance. Journal of Neuroscience.

[R14] Burr DC, Morrone MC, Vaina L (1998). Large receptive fields for optic flow direction in humans. Vision Research.

[R15] Burr DC, Santoro L (2001). Temporal integration of optic flow, measured by contrast and coherence thresholds. Vision Research.

[R16] Burr D, Thompson P (2011). Motion psychophysics: 1985-2010. Vision Research.

[R17] Challinor KL, Mather G (2010). A motion-energy model predicts the direction discrimination and MAE duration of two-stroke apparent motion at high and low retinal illuminance. Vision Research.

[R18] Chetverikov A, Jehee JFM (2019). Activity in human visual areas reflects the precision of motion perception. Journal of Vision.

[R19] Coletta NJ, Williams DR, Tiana CLM (1990). Consequences of spatial sampling for human motion perception. Vision Research.

[R20] Dakin SC, Turnbull PRK (2016). Similar contrast sensitivity functions measured using psychophysics and optokinetic nystagmus. Scientific Reports.

[R21] Derrington AM, Henning GB (1987). Errors in direction-of-motion discrimination with complex stimuli. Vision Research.

[R22] Edwards V, Giaschi D, Dougherty R, Edgell D, Bjornson B, Lyons C, Douglas R (2004). Psychophysical indexes of temporal processing abnormalities in children with dyslexia. Developmental Neuropsychology.

[R23] Giaschi D, Regan D (1997). The development of motion-defined figure-ground segregation in preschool and older children, using a letter-identification task. Optometry and Vision Science.

[R24] Glasser DM, Tadin D (2013). Reliable non-veridical perception of brief moving stimuli. Journal of Vision.

[R25] Glasser DM, Tadin D, Pack CC (2014). Motion reversal reveals mechanisms of perceptual suppression. Journal of Vision.

[R26] Green ML, Pratte MS (2020). Access to sensory uncertainty in global motion perception depends on the stimulus. Journal of Vision.

[R27] Gunn A, Cory E, Atkinson J, Braddick O, Wattam-Bell J, Guzzetta A, Cioni G (2002). Dorsal and ventral stream sensitivity in normal development and hemiplegia. Neuroreport.

[R28] Hadad BS, Maurer D, Lewis TL (2011). Long trajectory for the development of sensitivity to global and biological motion. Developmental Science.

[R29] Hayward J, Truong G, Partanen M, Giaschi D (2011). Effects of speed, age and amblyopia on the perception of motion-defined form. Vision Research.

[R30] Hwang BW, Schütz AC (2020). Idiosyncratic preferences in transparent motion and binocular rivalry are dissociable. Journal of Vision.

[R31] Manning C, Charman T, Pellicano E (2013). Processing slow and fast motion in children with autism spectrum conditions. Autism Research.

[R32] Manning C, Dakin SC, Tibber MS, Pellicano E (2014). Averaging, not internal noise, limits the development of coherent motion processing. Developmental Cognitive Neuroscience.

[R33] Manning C, Kaneshiro B, Kohler PJ, Duta M, Scerif G, Norcia AM (2019). Neural dynamics underlying coherent motion perception in children and adults. Developmental Cognitive Neuroscience.

[R34] Manning C, Wagenmakers E-J, Norcia AM, Scerif G, Boehm U (2021). Perceptual decision-making in children: Age-related differences and EEG correlates. Computational Brain & Behavior.

[R35] Meier K (2013). Resolving inconsistencies in the maturation of human global motion perception.

[R36] Meier K, Giaschi D (2014). The maturation of global motion perception depends on the spatial and temporal offsets of the stimulus. Vision Research.

[R37] Meier K, Giaschi D (2017). Effect of spatial and temporal stimulus parameters on the maturation of global motion perception. Vision Research.

[R38] Meier K, Giaschi D (2019). The effect of stimulus area on global motion thresholds in children and adults. Vision.

[R39] Meier K, Spering M, Giaschi D (2019). Fixation stability is not related to global motion deficits in amblyopia. Investigative Ophthalmology & Vision Science.

[R40] Meier K, Sum B, Giaschi D (2016). Global motion perception in children with amblyopia as a function of spatial and temporal stimulus parameters. Vision Research.

[R41] Milne E, Swettenham J, Hansen P, Campbell R, Jeffries H, Plaisted K (2002). High motion coherence thresholds in children with autism. Journal of Child Psychology and Psychiatry.

[R42] Moreno-Bote R, Shapiro A, Rinzel J, Rubin N (2008). Bi-stable depth ordering of superimposed moving gratings. Journal of Vision.

[R43] Morrone MC, Guzzetta A, Tinelli F, Tosetti M, Del Viva M, Montanaro D, Burr D, Cioni G (2008). Inversion of perceived direction of motion caused by spatial undersampling in two children with periventricular leukomalacia. Journal of Cognitive Neuroscience.

[R44] Narasimhan S, Giaschi D (2012). The effect of dot speed and density on the development of global motion perception. Vision Research.

[R45] Newsome WT, Paré EB (1988). A selective impairment of motion perception following lesions of the middle temporal visual area (MT). Journal of Neuroscience.

[R46] Nishida S (2011). Advancement of motion psychophysics: Review 2001-2010. Journal of Vision.

[R47] Nishida SY, Sato T (1992). Positive motion after-effect induced by bandpass-filtered random-dot kinematograms. Vision Research.

[R48] Parrish EE, Giaschi DE, Boden C, Dougherty R (2005). The maturation of form and motion perception in school age children. Vision Research.

[R49] Pellicano E, Gibson LY (2008). Investigating the functional integrity of the dorsal visual pathway in autism and dyslexia. Neuropsychologia.

[R50] Pilly PK, Seitz AR (2009). What a difference a parameter makes: A psychophysical comparison of random dot motion algorithms. Vision Research.

[R51] Qian N, Andersen RA, Adelson EH (1994). Transparent motion perception as detection of unbalanced signals. III. Modeling. Journal of Neuroscience.

[R52] Rideaux R, Welchman AE (2020). But still it moves: static image statistics underlie how we see motion. Journal of Neuroscience.

[R53] Scase MO, Braddick OJ, Raymond JE (1996). What is noise for the motion system?. Vision Research.

[R54] Schütz AC (2011). Motion transparency: Depth ordering and smooth pursuit eye movements. Journal of Vision.

[R55] Schütz AC, Braun DI, Movshon A, Gegenfurtner KR (2010). Does the noise matter? Effects of different kinematogram types on smooth pursuit eye movements and perception. Journal of Vision.

[R56] Serrano-Pedraza I, Goddard P, Derrington AM (2007). Evidence for reciprocal antagonism between motion sensors tuned to coarse and fine features. Journal of Vision.

[R57] Toffoli L, Scerif G, Snowling MJ, Norcia A, Manning C (2021). Global motion evoked potentials in autistic and dyslexic children: a cross-syndrome approach. Cortex.

[R58] Watamaniuk SNJ, Sekuler R (1992). Temporal and spatial integration in dynamic random-dot stimuli. Vision Research.

[R59] Williams DW, Sekuler R (1984). Coherent global motion percepts from stochastic local motions. Vision Research.

[R60] Yang Y, Blake R (1994). Broad tuning for spatial frequency of neural mechanisms underlying visual perception of coherent motion. Nature.

