# Supplementary material for: The reverse motion illusion in random dot motion displays and implications for understanding development
Source: J Illusion. Author manuscript; Available in PMC 2022 Feb 1. (PMC7612299; doi:10.47691/joi.v3.7916)
Supplement: Video caption [file EMS140862-supplement-Video_caption.pdf]

Video 1: Example of stimuli presented to participants in Meier & Giaschi (2014, 2017, 2019; Meier, Sum & Giaschi, 2016)

Example shows rightward motion stimuli at coherence levels between 100% and 0% using a white noise algorithm. Stimuli are presented for 600 ms. In this example, motion stimuli were created with a nominal Δt = 17 ms (dot positions are updated on every frame).

Video 2: Example of stimuli presented to participants in Meier & Giaschi (2014, 2017, 2019; Meier, Sum & Giaschi, 2016)

This example is identical to Video 1, except that stimuli were created with a nominal Δt = 50 ms (dot positions are updated once every 3 frames).

Video 3: Example of stimuli presented to participants in Narasimhan & Giaschi (2012)

Example shows rightward motion stimuli at coherence levels between 100% and 0% using a Brownian noise algorithm and a nominal density of 15 dots/deg^2. Stimuli are presented for 400 ms.

Video 4: Example of a stimulus presented to participants in Manning et al. (2019, 2021)

Example stimulus shows a period of random motion followed by directional motion, in which 75% of dots move coherently in an upwards direction.
